# Influence of response instructions and response format on applicant perceptions of a situational judgement test for medical school selection

**DOI:** 10.1186/s12909-018-1390-0

**Published:** 2018-11-26

**Authors:** Wendy E. De Leng, Karen M. Stegers-Jager, Marise Ph. Born, Axel P. N. Themmen

**Affiliations:** 1 Institute of Medical Education Research Rotterdam, Erasmus MC, IMERR, Room AE-227, PO Box 2040, 3000CA Rotterdam, the Netherlands; 2 Institute of Psychology, Erasmus University Rotterdam, PO Box 1738, 3000DR Rotterdam, the Netherlands

**Keywords:** Situational judgement test, Medical school selection, Applicant perceptions, Response instructions, Response format

## Abstract

**Background:**

This study examined the influence of two Situational Judgement Test (SJT) design features (response instructions and response format) on applicant perceptions. Additionally, we investigated demographic subgroup differences in applicant perceptions of an SJT.

**Methods:**

Medical school applicants (*N* = 372) responded to an online survey on applicant perceptions, including a description and two example items of an SJT. Respondents randomly received one of four SJT versions (should do-rating, should do-pick-one, would do-rating, would do-pick-one). They rated overall favourability and items on four procedural justice factors (face validity, applicant differentiation, study relatedness and chance to perform) and ease-of-cheating. Additionally, applicant perceptions were compared for subgroups based on gender, ethnic background and first-generation university status.

**Results:**

Applicants rated would-do instructions as easier to cheat than should-do instructions. Rating formats received more favourable judgements than pick-one formats on applicant differentiation, study-relatedness, chance to perform and ease of cheating. No significant main effect for demographic subgroup on applicant perceptions was found, but significant interaction effects showed that certain subgroups might have more pronounced preferences for certain SJT design features. Specifically, ethnic minority applicants – but not ethnic majority applicants – showed greater preference for should-do than would-do instructions. Additionally, first-generation university students – but not non-first-generation university students – were more favourable of rating formats than of pick-one formats.

**Conclusions:**

Findings indicate that changing SJT design features may positively affect applicant perceptions by promoting procedural justice factors and reducing perceived ease of cheating and that response instructions and response format can increase the attractiveness of SJTs for minority applicants.

**Electronic supplementary material:**

The online version of this article (10.1186/s12909-018-1390-0) contains supplementary material, which is available to authorized users.

## Background

An increasing number of medical schools implement a Situational Judgement Test (SJT) in their admission procedures [1–4]. The growing popularity of the SJT is a result of the test’s psychometric qualities, in terms of its predictive validity, incremental validity and low adverse impact, from the perspective of medical school admission committees [5]. Yet, the quality of an SJT should also be investigated from the perspective of medical school applicants, since applicant perceptions may influence test-taking motivation, test performance and applicant withdrawal [6, 7]. Furthermore, minority applicants may hold more negative applicant perceptions [8], which could lead to adverse impact through diminished test-taking motivation and test performance. The current study examines the influence of two SJT design features, namely response instructions and response format, on applicant perceptions. Additionally, the perceptions of the SJT are compared for applicants belonging to different demographic subgroups.

SJTs require respondents to judge the appropriateness of responses to challenging situations [9]. The situations are contextualised to the setting for which an individual applies, such as medical school. In general, SJTs are added to admission procedures for the measurement of noncognitive attributes, for instance integrity and interpersonal skills [10]. Prior research has demonstrated that SJTs have predictive validity for future medical school performance [11], that they have incremental validity over traditional cognitive admission instruments [12] and smaller ethnic and socioeconomic subgroup differences than cognitive admission tests [13, 14].

In addition to these psychometric findings, several studies have demonstrated that medical school applicants hold favourable perceptions of SJTs [11, 15–17]. Moreover, some studies indicated that SJTs are perceived more positively than cognitive admission tests [11, 16]. Favourable perceptions of SJTs are likely caused by the test content, which is closely related to the criterion domain for which an individual applies [18]. Furthermore, previous research demonstrated that certain SJT features might affect applicants’ perceptions [19]. For example, Chan and Schmitt [6] and Kanning et al. [20] found that applicants perceived the same SJT more positively when it was administered in a video-based format than in a text-based format. Additionally, Neal et al. [21] showed that medical students felt that an SJT with a short-answer-question or an interview response format would better reflect their future behaviour as a junior doctor than a ranked-order or a single-best-answer response format. Response formats using short-answer or interview questions received the most favourable ratings, probably because applicants believe these formats provide a good opportunity to demonstrate their knowledge, skills and abilities [22]. No prior research has examined the influence of SJT response instructions on applicant perceptions.

The importance of applicant perceptions is evidenced by the influence of these perceptions on test-taking motivation and test performance [6] and possible applicant withdrawal [7]. Additionally, prior research indicated that applicant perceptions might differ across demographic subgroups. For example, ethnic minorities tend to perceive selection procedures at large more negatively than ethnic majorities [6, 8], possibly due to differences in cultural values and beliefs on testing [23] or by perceptions of stereotype threat [24], which refers to impaired test performance caused by the salience of a negative stereotype [25]. More negative perceptions of the admission procedure might reduce test performance through decreased test-taking motivation [26]. Unfavourable perceptions of the admission process among ethnic minorities might also result in disproportionally more withdrawal from the admission procedure among ethnic minority applicants [7]. Thus, if minority applicants – based on either gender, ethnic or socioeconomic background – perceive an admission procedure more negatively than majority applicants, they might also be less motivated to perform well or more inclined to withdraw from the admission procedure. Consequently, more negative applicant perceptions among minority applicants may lead to adverse impact. It is therefore crucial to examine which design features of an SJT reduce subgroup differences in applicant perceptions. We are not aware of previous studies that have focused on how response instructions and response format may influence subgroup differences in applicant perceptions of an SJT.

The dominant theoretical framework on applicant perceptions is the organisational justice theory [27]. This theory has been applied to studies on applicant perceptions of selection practices for postgraduate medical training [28] and of admission methods in higher education [29]. The organisational justice theory encompasses distributive justice, that is the fairness of the distribution of desired outcomes (e.g. admission spots in medical school) and procedural justice, referring to the fairness of procedures used to allocate desired outcomes [27]. In the model of applicant reactions proposed by Gilliland, procedural justice perceptions are influenced by the formal characteristics of the selection system, like job relatedness and opportunity to perform. According to the organisational justice model, formal characteristics are influenced by test type. Therefore, the formal characteristics component was used to study the influence of SJT design features on applicant perceptions.

The aim of the present study is two-fold. Firstly, we examined the effect of the response instructions (i.e. should do or would do) and the response format (i.e. pick-one or rating) of an SJT on applicant perceptions. The influence of response instructions was examined because previous research showed that SJTs with should-do instructions are less susceptible to faking than SJTs with would-do instructions [30]. Additionally, admission procedures that are perceived as more difficult to fake receive more favourable applicant perceptions [31, 32]. Therefore, we hypothesised that applicants have more positive perceptions of an SJT using should-do instructions than an SJT using would-do instructions. The influence of response format on applicant perceptions of an SJT was previously investigated by Neal et al. [21]. However, these researchers did not include a rating format in their investigation, even though this response format is commonly used by SJTs [6, 17]. We expected the pick-one format to receive more favourable applicant perceptions than the rating format because applicants – at least in Western cultures – are more familiar with the use of pick-one (i.e. multiple-choice) formats in college admission, such as in cognitive ability tests [28]. Additionally, we assumed that applicants associate rating formats with self-report measures, which are prone to faking and therefore perceived less favourably.

Secondly, to determine if SJTs are perceived differently by minority applicants than majority applicants, we examined the influence of demographic variables (i.e. gender, ethnic background and first-generation university status) on applicant perceptions. Based on previous research, we hypothesised to find no gender differences in applicant perceptions [29, 33]. The meta-analysis of Hausknecht et al. [33] indicated that the correlation between ethnic background and applicant perceptions was near zero. However, Chan [23] found that among a US sample the predictive validity perceptions of a cognitive ability test – but not of a personality test – were significantly more favourable for White than for Black examinees. Since SJTs – like personality tests – focus on noncognitive attributes, we expected no ethnic differences in applicant perceptions. Prior research on subgroup differences in applicant perceptions has mainly focused on gender and ethnicity, but not on socioeconomic characteristics such as the educational level of the applicant’s parents. Therefore, we pose the following research question: do applicant perceptions of an SJT differ across subgroups from different socioeconomic backgrounds?

## Methods

### Setting and procedure

This study was conducted at a Dutch medical school, whose admission procedure consisted of three equally-weighted parts: i) pre-university grade point average (pu-GPA), ii) extracurricular activities and iii) performance on three cognitive tests during an on-site testing day. Applicants with a pu-GPA ≥ 7.5 (on a scale from 1 (low performance) to 10 (high performance)) were directly admitted. The applicants of the 2018 admission procedure comprised the sample of this study. After the on-site testing day but before the applicants received the selection decision, applicants were invited to participate in an online survey on applicant perceptions. Participation in the survey was voluntary. Applicants were informed about the aim of the survey and that their answers would not influence the selection decision. Applicants gave informed consent before they were navigated to the survey. The data in this study were processed anonymously.

### Survey

The online survey started with a questionnaire on the applicants’ demographic characteristics. The demographic questions were administered online for the applicants with pu-GPA ≥ 7.5 and on-site for other responders. Applicants were categorised as first-generation university student, if both their parents had not attended university. The ethnic background of the applicants was categorised as Dutch if both parents were born in the Netherlands, as non-Western if at least one parent was born in Africa, Asia or South-America, or as Western if at least one parent was born in Europe (but not in the Netherlands), North-America or Oceania [34]. The applicants’ gender was retrieved from the student administration system.

The second part of the survey covered applicant perceptions. Applicant perceptions were measured using seven items. Firstly, overall process favourability was assessed using two items: perceived predictive validity and perceived fairness [35]. Steiner and Gilliland [35] reported a coefficient alpha of .73 for the two process favourability items. Secondly, four items were administered measuring formal characteristics of the procedural justice dimension: i) face validity, ii) applicant differentiation [35], iii) study relatedness and iv) chance to perform [36]. These items were selected because previous research demonstrated the influence of these formal characteristics on process favourability [22, 29, 33]. Finally, one item measuring ease of cheating [29] was added because a prior meta-analysis showed that ease of cheating/difficulty to fake has a negative influence on applicant perceptions [32]. Each item was judged on a seven-point anchored rating scale. The items and rating scales are depicted in Additional file 1.

The survey asked respondents to answer the seven applicant perception items separately for eleven admission instruments (CV, motivation letter, pre-university GPA, cognitive capacity test, skills test, curriculum sample test, personality questionnaire, interview, weighted lottery, unweighted lottery and SJT). The order in which the admission instruments were presented to the respondents was randomised.

Survey respondents received a short description of the SJT followed by two examples of SJT items. These example items were identical, with the exception of two design features that were manipulated. Firstly, the response instructions: the example items asked either which response should be given in the described situation (i.e. should do) or which response the respondents were most likely to perform (i.e. would do). Secondly, the response format: the example items had to be judged either by rating each separate response option (i.e. rating format) or by picking out the best response option (i.e. pick-one). In total, there were four versions of the SJT example items (i.e. should do-rating, should do-pick-one, would do-rating, would do-pick-one). Each respondent randomly received two SJT example items representing one of the four versions.

### Statistical analyses

Two-way ANOVAs were used to examine the influence of SJT response instructions (should do versus would do) and SJT response format (rating versus pick one) on process favourability, the four procedural justice items (i.e. face validity, applicant differentiation, study relatedness, chance to perform) and ease of cheating. Main and interaction effects were examined. Pu-GPA ≥ 7.5 status (i.e. directly admitted) was included in the analyses as a control variable. Partial eta-squared was used to examine the effect sizes, where *η*_*p*_^*2*^ = .01, *η*_*p*_^*2*^ = .06 and *η*_*p*_^*2*^ = .14 indicates a small, medium and large effect, respectively [37].

ANOVAs were used to examine subgroup differences (based on gender, ethnic background and first-generation university status) on the applicant perception items. Pu-GPA ≥ 7.5 status was again included as a control variable. In addition, the demographic variables were investigated in relation to the SJT design features by examining if the subgroup variables had an interaction effect with either the response instructions or the response format. Partial eta-squared was used to examine the effect size.

## Results

### Participants

In total, 872 applicants were invited to participate in the survey. Three-hundred seventy-two applicants responded to the survey (response rate = 42.7%). The average age of this group was 18.35 years (*SD* = 1.19) and 75.3% were women. Among the 372 respondents, 26.6% were first-generation university students, 70.2% had a Dutch ethnic background, 21.5% had a non-Western ethnic background, 8.3% had a Western ethnic background and 38.7% were directly admitted to medical school (i.e. pu-GPA ≥ 7.5). The group of respondents was significantly younger (18.35 vs. 18.64 years, *t*(870) = 3.39, *p* = .001, *d* = 0.24) and consisted of significantly more women (75.3% vs. 65.7%, *X*^*2*^(1) = 8.91, *p* = .003, *ϕ* = 0.10) than the group of non-respondents, but the effect sizes were small. Respondents and non-respondents were comparable with respect to first-generation university status (*X*^*2*^(1) = 1.30, *p* = .254) and ethnic background (*X*^*2*^(2) = 2.47, *p* = .291).

### Applicant perception items

The alpha coefficients of the two process favourability items (i.e. perceived predictive validity and perceived fairness) indicated sufficient to good internal consistency (should do-rating: *α* = .66, should do-pick-one: *α* = .75, would do-rating: *α* = .72, would do-pick-one: *α* = .90). The intercorrelations between the process favourability score (i.e. average of the two process favourability items) and the other applicant perception items are depicted in Table 1. Intercorrelations were controlled for pu-GPA ≥ 7.5 status (i.e. directly admitted). All intercorrelations were statistically significant. The correlations between process favourability and the procedural justice items were all above .6 (large effect size). As expected, the ease-of-cheating item correlated significantly and negatively with process favourability, but the effect size was smaller (*r* = −.20).

**Table 1:** Intercorrelations between overall process favourability and the other applicant perception items

	1.	2.	3.	4.	5.
1. Process favourability					
2. Face validity	.76				
3. Applicant differentiation	.67	.69			
4. Study relatedness	.62	.64	.62		
5. Chance to perform	.63	.59	.67	.63	
6. Ease of cheating	−.20	−.24	−.20	−.26	−.24

*Note.* All correlations are significant, *p* < .01 (two-tailed) Correlations are controlled for pu-GPA ≥ 7.5 status (i.e. directly admitted)

### Preliminary analysis: Comparison to other admission methods

Prior to the main analyses, we compared the overall process favourability of the SJT to the other admission methods included in the online survey, in order to determine if the SJT was perceived more or less positively than the other admission methods (Table 2). Repeated-measures ANOVAs were used to examine the differences in process favourability between the SJT and each of the other admission methods. We controlled for pu-GPA ≥ 7.5 status by including it as a between-subjects factor. The average process favourability rating (on a seven-point scale) ranged between 3.21 (unweighted lottery) and 5.29 (interview). The SJT was judged significantly more favourable than pu-GPA (*F*(1, 364) = 7.04, *p* = .008, *η*_*p*_^*2*^ = .02), a personality questionnaire (*F*(1, 365) = 17.89, *p* < .001, *η*_*p*_^*2*^ = .05), weighted lottery (*F*(1, 365) = 67.07, *p* < .001, *η*_*p*_^*2*^ = .16) and unweighted lottery (*F*(1, 366) = 114.31, *p* < .001, *η*_*p*_^*2*^ = .24) and significantly less favourable than a motivation letter (*F*(1, 365) = 22.11, *p* < .001, *η*_*p*_^*2*^ = .06), cognitive capacity test (*F*(1, 363) = 17.68, *p* < .001, *η*_*p*_^*2*^ = .05), skills test (*F*(1, 364) = 87.78, *p* < .001, *η*_*p*_^*2*^ = .19), curriculum sample test (*F*(1, 367) = 105.17, *p* < .001, *η*_*p*_^*2*^ = .22) and an interview (*F*(1, 364) = 119.50, *p* < .001, *η*_*p*_^*2*^ = .25). CV was judged as equally favourable as the SJT. Thus, among the other admission methods included in the online survey, the SJT takes a middle position with respect to overall process favourability.

**Table 2:** Comparison of the Situational Judgement Test with the other admission methods on process favourability

	Process favourability
Situational Judgement Test	4.39 (1.28)
Curriculum vitae	4.44 (1.42)
Motivation letter	**4.76 (1.22)**
Pre-university GPA	**3.93 (1.46)**
Cognitive capacity test	**4.69 (1.15)**
Skills test	**5.11 (1.09)**
Curriculum sample test	**5.20 (1.05)**
Personality questionnaire	**4.02 (1.26)**
Interview	**5.29 (1.20)**
Weighted lottery	**3.40 (1.55)**
Unweighted lottery	**3.21 (1.82)**

*Note.* GPA = grade point average Bold averages indicate a significant difference from the average judgement of process favourability for the Situational Judgement Test (repeated-measures ANOVA with GPA ≥ 7.5 as between-subjects factor, *p* < .01)

### Response instructions and format

**Fig. 1:**
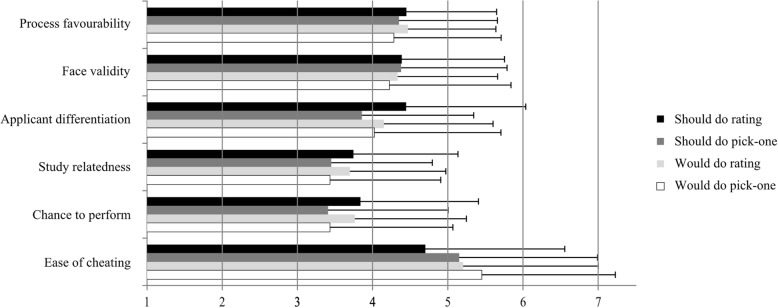
Process favourability and judgements on the other applicant perception items for the four SJT versions. Error bars reflect standard deviations

Applicant perceptions of the four SJT versions are depicted in Fig. 1. The mean and standard deviations corresponding to Fig. 1 can be found in Additional file 2. A significant main effect of response format was found on the applicant differentiation item (*F*(1, 362) = 4.08, *p* = .044, *η*^*2*^ = .01) with a more positive judgement for the rating format (*M* = 4.30, *SD* = 1.53) than for the pick-one format (*M* = 3.94, *SD* = 1.59). Response format also had a significant influence on the study-relatedness item (*F*(1, 362) = 4.23, *p* = .040, *η*^*2*^ = .01), again indicating more favourable perceptions for the rating format (*M* = 3.73, *SD* = 1.33) than for the pick-one format (*M* = 3.44, *SD* = 1.41). The rating format (*M* = 3.81, *SD* = 1.52) was also judged significantly more favourable than the pick-one format (*M* = 3.42, *SD* = 1.61) on the chance-to-perform item (*F*(1, 361) = 5.16, *p* = .024, *η*^*2*^ = .01). Finally, the pick-one format (*M* = 5.31, *SD* = 1.81) was judged as significantly easier to cheat than the rating format (*M* = 4.94, *SD* = 1.84; *F*(1, 362) = 5.29, *p* = .022, *η*^*2*^ = .01). Overall, an SJT with a rating response format was rated more favourably than an SJT with a pick-one format on applicant differentiation, study-relatedness, chance to perform and ease of cheating. Thus, the rating format was – in contrast to our hypothesis – judged more favourable than the pick-one format. Finally, response instructions had a significant main effect on the ease-of-cheating item (*F*(1, 362) = 4.53, *p* = .034, *η*^*2*^ = .01) with the would-do instructions (*M* = 5.33, *SD* = 1.79) judged as easier to cheat than the should-do instructions (*M* = 4.92, *SD* = 1.86). With regard to our hypothesis, no differences between should-do and would-do instructions were found for the overall process favourability, but should-do instructions were judged as more difficult to cheat than would-do instructions. Two-way ANOVAs revealed no significant interaction effects between response instructions and response format.

### Subgroup differences

The demographic subgroup differences in applicant perceptions are shown in Table 3. No significant main effects were found for gender, ethnic background or first-generation university status on the judgements of process favourability, the procedural justice factors and ease of cheating. However, significant interaction effects between subgroup and either response instructions or response format were found. Demographic subgroup differences for the four separate SJT versions are depicted in Additional file 2.

**Table 3:** Average judgement on process favourability and the other applicant perception items for the different subgroups

		Gender		First-generation university		Ethnic background		
	Overall	M	W	Yes	No	Dutch	NW	W
Process favourability	4.39 (1.28)	4.38 (1.34)	4.39 (1.26)	4.47 (1.29)	4.36 (1.23)	4.41 (1.26)	4.35 (1.25)	1.36 (1.44)
Face validity	4.33 (1.43)	4.28 (1.53)	4.34 (1.40)	4.56 (1.31)	4.21 (1.47)	4.27 (1.43)	4.49 (1.50)	4.10 (1.32)
Applicant differentiation	4.12 (1.57)	4.04 (1.74)	4.15 (1.51)	4.10 (1.61)	4.12 (1.56)	4.04 (1.54)	4.35 (1.66)	4.17 (1.56)
Study relatedness	3.58 (1.38)	3.55 (1.52)	3.59 (1.33)	3.70 (1.33)	3.55 (1.37)	3.57 (1.32)	3.75 (1.47)	3.45 (1.45)
Chance to perform	3.61 (1.58)	3.64 (1.66)	3.60 (1.56)	3.74 (1.61)	3.54 (1.55)	3.56 (1.57)	3.83 (1.57)	3.38 (1.50)
Ease of cheating	5.13 (1.83)	5.22 (1.89)	5.10 (1.81)	5.05 (1.73)	5.20 (1.84)	5.22 (1.83)	5.11 (1.76)	4.86 (1.85)

*Note. M* Men, *W* Women, *NW* non-Western, *W* Western Standard deviations between brackets

Gender and response format had a significant interaction effect on the applicant differentiation item (*F*(1, 362) = 4.80, *p* = .029, *η*^*2*^ = .01) and the study-relatedness item (*F*(1, 362) = 7.64, *p* = .006, *η*^*2*^ = .02). The more positive judgement of the rating format than the pick-one format was stronger for men than for women on the applicant differentiation item (*d* = 0.46 vs. *d* = 0.15) and on the study-relatedness item (*d* = 0.61 vs. *d* = 0.08). Ethnic background and response instructions had a significant interaction effect on process favourability (*F*(2, 336) = 4.42, *p* = .013, *η*_*p*_^*2*^ = .03), the face validity item (*F*(2, 333) = 3.61, *p* = .028, *η*_*p*_^*2*^ = .02) and the study-relatedness item (*F*(2, 335) = 3.10, *p* = .046, *η*_*p*_^*2*^ = .02). For applicants from a Dutch background, should-do and would-do instructions were rated similarly on process favourability (*d* = 0.03), the face validity item (*d* = 0.04) and the study-relatedness item (*d* = 0.02). In contrast, applicants from a non-Western background were more positive on should-do than would-do instructions (process favourability: *d* = 0.36; face validity: *d* = 0.41; study relatedness: *d* = 0.27), whereas applicants from a Western background were more positive on would-do than should-do instructions (process favourability: *d* = − 0.42; face validity: *d* = − 0.12; study relatedness: *d* = − 0.36). First-generation university status and response format had a significant interaction effect on process favourability (*F*(1, 341) = 5.23, *p* = .023, *η*^*2*^ = .02), the face validity item (*F*(1, 338) = 9.80, *p* = .002, *η*^*2*^ = .03) and the applicant differentiation item (*F*(1, 340) = 4.25, *p* = .040, *η*^*2*^ = .01). First-generation university students judged an SJT with a rating format more favourably than an SJT with a pick-one format on process favourability (*d* = 0.45), the face validity item (*d* = 0.51) and the applicant differentiation item (*d* = 0.42). In contrast, for non-first-generation university students, both response formats were judged similarly on process favourability (*d* = 0.05), the face validity item (*d* = − 0.15) and the applicant differentiation item (*d* = 0.13). Thus, as stated by our hypotheses, subgroups based on gender and ethnic background did not significantly differ in their applicant perceptions of an SJT. Regarding our research question, we found no significant difference in applicant perceptions between the subgroups based on first-generation university status. Nonetheless, the findings do indicate that subgroups might differ in their preference for certain SJT design features.

## Discussion

The present study indicates that response format and – to a lesser extent – response instructions influence applicants’ perceptions of an SJT. The results show that asking applicants to rate each separate response option leads to more favourable perceptions of an SJT than asking applicants to pick one of the responses as the best option. Additionally, when instructed to respond according to what they would actually do in the described situation, applicants perceive an SJT as easier to cheat than when instructed to respond according to what should be done in the described situation. The applicant subgroups based on gender, ethnic background or first-generation university status were comparable regarding their perceptions of the SJT. However, our results do show that applicants from a non-Western ethnic background hold more positive perceptions of an SJT with should-do instructions than of an SJT with would-do instructions. On the contrary, applicants from a Western ethnic background appear to be more positive about an SJT with would-do instructions than an SJT with should-do instructions. Additionally, men and first-generation university students perceive an SJT with a rating response format more favourably than an SJT with a pick-one response format.

Response instructions had a significant influence on the perceived ease of cheating, indicating that should-do instructions are not only statistically less susceptible to faking [38, 39], but are also perceived as more difficult to fake than would-do instructions. Previous research has shown that applicants’ perceptions of a test do not always correspond to the actual psychometric qualities of that test [40]. For example, Chan [23] found that personality tests were perceived as more predictive than cognitive ability tests, whereas empirical studies show that cognitive ability tests are more predictive than personality tests. Apparently, ease of cheating is more obvious to applicants than the predictive value of a test and might therefore provide a more effective means to enhance applicant perceptions. Response instructions had no significant effect on the overall process favourability of the SJT. Nevertheless, the negative association between process favourability and ease of cheating indicates that applicant perceptions may be enhanced by reducing the SJT’s susceptibility to faking.

In contrast to our hypothesis, a rating response format was perceived more positively than a pick-on response format on three of the procedural justice factors and ease of cheating. We had expected applicants to be more positive on pick-one formats because applicants are more familiar with this response format in traditional multiple-choice admission tests [28, 41] and because rating formats are commonly used by easier-to-fake self-report measures. However, the results of this study indicate that applicants perceive rating formats as a better measure to differentiate between applicants, as more strongly related to medical school, as a better means to show skills and abilities and as more difficult to cheat than pick-one formats. Possible explanations for this finding are that rating formats provide applicants the possibility to give more nuanced responses and allow applicants to give a rating of all response options. The challenging situations described in SJTs may be solved using multiple approaches, causing pick-one formats to be perceived as unrealistic [42]. Response formats that allow for more nuanced answers might better fit the dilemma-like nature of SJTs. Likewise, medical students preferred an SJT with a short-answer-question format over an SJT with a single-best-answer format [21]. Unlike our expectations, the rating format was not judged as easier to cheat than the pick-one format. Apparently, when used in SJTs, rating formats are not associated with the negative characteristics of self-report measures in a selection context. More favourable perceptions of the rating format are desirable as previous research has demonstrated that rating formats are superior to other response formats on a variety of psychometric outcomes [43].

Applicant perceptions did not differ across subgroups based on gender, ethnic background and first-generation university status. The absence of subgroup differences is in line with findings of previous studies [29, 33, 40]. Nevertheless, the significant interaction effects do indicate that certain subgroups might have more pronounced preferences for certain SJT design features. Specifically, men seem to perceive rating formats more positively than pick-one formats regarding applicant differentiation and study relatedness. Prior research on cognitive ability tests showed that open-ended response formats resulted in less gender differences in test performance than multiple-choice response formats [44]. Arthur et al. [43] found that the gender difference in an SJT score was larger for a ranking format than for a rating format and most/least-effective format. This interaction effect between gender and response format on test performance might translate into a gender-response format interaction on applicant perceptions. More research is required to unravel this interaction effect.

Non-Western ethnic minority applicants appear to be more positive on should-do than would-do instructions. Although a previous study demonstrated that the administration method (paper-and-pencil vs. video-based) affected the Black-White difference in applicants perceptions of an SJT [6], this is the first study showing that response instructions might also affect ethnic differences in applicant perceptions of an SJT. McDaniel et al. [30] demonstrated that SJTs with knowledge instructions (i.e. should do) had higher correlations with cognitive ability test, whereas SJTs with behavioural tendency instructions (i.e. would do) had higher correlations with personality. Applicants from a non-Western background might feel that knowledge-based tests are more face valid and stronger related to medical school than personality-based tests and therefore perceive should-do instructions more favourably. Another possible explanation for more positive perceptions of should-do instructions among non-Western ethnic minority applicants might be found in differences between individualistic and collectivistic cultures [45]. We presume that non-Western minority applicants may have a stronger collectivistic cultural orientation than majority applicants and might therefore be more comfortable to judge the SJT response options according to the group norms instead of according to their own individual norms [46]. Additionally, results seem to indicate that Western ethnic minority applicants are more favourable of would-do than should-do instructions. However, the sample size of the Western minority applicant group was very small, making it difficult to draw strong conclusions from this finding. Future research is necessary to replicate these findings and to examine potential explanations.

First-generation university students perceive rating formats more positively than pick-one formats. It appears that applicants from a low socio-economic background have a stronger preference for response formats that permit more nuanced responses than applicants from a high socio-economic background. A possible explanation might be that applicants whose parents did not attend university have more negative test-taking attitudes on traditional formats of testing. SJTs with pick-one formats might be more strongly associated with traditional tests and therefore receive more negative perceptions. Nevertheless, prior research on demographic differences in applicant perceptions has mainly focused on gender and ethnic background. Thus, future research should take into account socioeconomic background when examining subgroup differences in applicant perceptions and should examine why first-generation university students are more favourable of rating formats.

### Practical implications

Our findings present two practical implications for medical school admission committees which use an SJT and are concerned with the applicant perceptions of that SJT. Firstly, using should-do instructions as opposed to would-do instructions increases the SJT’s favourability among ethnic minority applicants. Secondly, men and first-generation university students perceived an SJT with a rating format more positively than an SJT with a pick-one format. Moreover, applicant perceptions did not differ between the two response instructions and the two response formats for the majority applicants. Therefore, using these SJT design features to positively influence applicant perceptions among minority applicants does not lead to unfavourable perceptions among majority applicants.

### Limitations and directions for future research

Although applicant perceptions in this study are solely based on a short description and two example items of an SJT, minor changes in the example items led to significant differences in applicant perceptions. Nonetheless, future research should assess the applicants’ perspective after completing a full version of an SJT, preferably one that is used for the actual selection into medical school, to obtain a more thorough picture of the influence of changing the SJT design features on applicant perceptions.

Prior research has indicated that applicant perceptions may influence applicant behaviour (e.g. applicant withdrawal, recommendations to others) [7, 47]. However, the present study is limited to examining the influence of SJT design features on applicant perceptions. The behavioural consequences of positive or negative applicant perceptions of an SJT need to be addressed in future research.

In general, the average judgements on the applicant perception items were situated close to the midpoints of the rating scales. Additionally, the SJT was judged significantly less favourable than five of the ten other admission methods included in the online survey (i.e. motivation letter, cognitive capacity test, skills test, curriculum sample test and interview). Even though this study demonstrated that changing the design features of an SJT may enhance applicant perceptions, future research is advised to examine the influence of other SJT characteristics that may positively affect perceptions of SJTs.

Finally, perceptions of procedural justice are not only determined by the formal characteristics of the admission procedure, but also by the treatment of applicants and the explanations of admission procedures and decisions (i.e. interactional justice) [27]. Enhancing applicants’ perceptions of an SJT must be accompanied by devoting attention to these other aspects of the medical school admission procedure.

## Conclusions

The applicant’s perspective on the use of SJTs in medical school admission procedures should not be underestimated, because applicant perceptions might influence test-taking motivation, test performance and applicant withdrawal. The current study demonstrated that changing the response format of an SJT may positively affect applicant perceptions through advancing the procedural justice factors of applicant differentiation, study relatedness and chance to perform and by reducing the perceived ease of cheating. Additionally, applicant perceptions may be altered by using response instructions that are less susceptible to faking. Finally, this study indicated that certain design features may lead to more favourable perceptions of an SJT among minority applicants, presenting another potential measure for promoting widening access to medical school.

## Additional files

Additional file 1: SJT applicant perceptions: Microsoft Word Document (.docx): Applicant perception items: The seven items on applicants perceptions that were administered for the four versions of the SJT. (DOCX 14 kb)

Additional file 2: SJT applicant perceptions: Microsoft Word Document (.docx): Mean and standard deviations for the four SJT versions: Means (and standard deviations) for process favourability and the other applicant perception items for the four SJT versions and for each subgroup. (DOCX 21 kb)

## Abbreviations

ANOVA: Analysis of Variance
CV: Curriculum Vitae
GPA: Grade Point Average
Pu-GPA: pre-university Grade Point Average
SJT: Situational Judgement Test

## References

[1] Dore KL, Reiter HI, Eva KW, Krueger S, Scriven E, Siu E, Hilsden S, Thoman J, Norman GR (2009). Extending the interview to all medical school candidates—computer-based multiple sample evaluation of noncognitive skills (CMSENS). Acad Med.

[2] Fröhlich M, Kahmann J, Kadmon M (2017). Development and psychometric examination of a German video-based situational judgment test for social competencies in medical school applicants. Int J Sel Assess.

[3] Schripsema NR, van Trigt AM, Borleffs JCC, Cohen-Schotanus J (2017). Impact of vocational interests, previous academic experience, gender and age on situational judgement test performance. Adv Health Sci Educ.

[4] Patterson F, Cousans F, Edwards H, Rosselli A, Nicholson S, Wright B (2017). The predictive validity of a text-based situational judgment test in undergraduate medical and dental school admissions. Acad Med.

[5] Patterson F, Zibarras L, Ashworth V (2016). Situational judgement tests in medical education and training: research, theory and practice: AMEE guide no. 100. Med Teach.

[6] Chan D, Schmitt N (1997). Video-based versus paper-and-pencil method of assessment in situational judgment tests: subgroup differences in test performance and face validity perceptions. J Appl Psychol.

[7] Schmit MJ, Ryan AM (1997). Applicant withdrawal: the role of test-taking attitudes and racial differences. Pers Psychol.

[8] Ryan AM, Sacco JM, McFarland LA, Kriska SD (2000). Applicant self-selection: correlates of withdrawal from a multiple hurdle process. J Appl Psychol.

[9] Weekley JA, Ployhart RE (2006). Situational judgment tests: Theory, measurement, and application.

[10] Patterson F, Ashworth V, Zibarras L, Coan P, Kerrin M, O’Neill P (2012). Evaluations of situational judgement tests to assess non-academic attributes in selection. Med Educ.

[11] Lievens F (2013). Adjusting medical school admission: assessing interpersonal skills using situational judgement tests. Med Educ.

[12] Koczwara A, Patterson F, Zibarras L, Kerrin M, Irish B, Wilkinson M (2012). Evaluating cognitive ability, knowledge tests and situational judgement tests for postgraduate selection. Med Educ.

[13] Oswald FL, Schmitt N, Kim BH, Ramsay LJ, Gillespie MA (2004). Developing a biodata measure and situational judgment inventory as predictors of college student performance. J App Psychol.

[14] Lievens F, Patterson F, Corstjens J, Martin S, Nicholson S (2016). Widening access in selection using situational judgement tests: evidence from the UKCAT. Med Educ.

[15] Lievens F, Sackett PR (2006). Video-based versus written situational judgment tests: a comparison in terms of predictive validity. J Appl Psychol.

[16] Luschin-Ebengreuth M, Dimai HP, Ithaler D, Neges HM, Reibnegger G (2015). Situational judgment test as an additional tool in a medical admission test: an observational investigation. BMC Res Notes.

[17] Husbands A, Rodgerson MJ, Dowell J, Patterson F (2015). Evaluating the validity of an integrity-based situational judgement test for medical school admissions. BMC Med Educ.

[18] Lievens F, Peeters H, Schollaert E (2008). Situational judgment tests: a review of recent research. Pers Rev.

[19] Bauer TN, Truxillo DM, Weekley JA, Ployhart RE (2006). Applicant reactions to situational judgment tests: research and related practical issues. Situational judgment tests: theory, measurement, and application.

[20] Kanning UP, Grewe K, Hollenberg S, Hadouch M (2006). From the Subjects' point of view. Eur J Psychol Assess.

[21] Neal GEH, Oram RC, Bacon AJ (2017). What do students think about the situational judgment test?. Med Teach.

[22] Schleicher DJ, Venkataramani V, Morgeson FP, Campion MA (2006). So you didn't get the job… now what do you think? Examining opportunity-to-perform fairness perceptions. Pers Psychol.

[23] Chan D (1997). Racial subgroup differences in predictive validity perceptions on personality and cognitive ability tests. J Appl Psychol..

[24] Ployhart RE, Ziegert JC, McFarland LA (2003). Understanding racial differences on cognitive ability tests in selection contexts: an integration of stereotype threat and applicant reactions research. Hum Perform.

[25] Steele CM, Aronson J (1995). Stereotype threat and the intellectual test performance of African Americans. J Pers Soc Psychol.

[26] Chan D, Schmitt N, DeShon RP, Clause CS, Delbridge K (1997). Reactions to cognitive ability tests: the relationships between race, test performance, face validity perceptions, and test-taking motivation. J Appl Psychol..

[27] Gilliland SW (1993). The perceived fairness of selection systems: an organizational justice perspective. Acad Manag Rev.

[28] Patterson F, Zibarras L, Carr V, Irish B, Gregory S (2011). Evaluating candidate reactions to selection practices using organisational justice theory. Med Educ.

[29] Niessen ASM, Meijer RR, Tendeiro JN (2017). Applying organizational justice theory to admission into higher education: admission from a student perspective. Int J Sel Assess.

[30] McDaniel MA, Hartman NS, Whetzel DL, Grubb W (2007). Situational judgment tests, response instructions, and validity: a meta-analysis. Pers Psychol.

[31] Schreurs B, Derous E, Proost K, Notelaers G, Witte KD (2008). Applicant selection expectations: validating a multidimensional measure in the military. Int J Sel Assess.

[32] Uggerslev KL, Fassina NE, Kraichy D (2012). Recruiting through the stages: a meta-analytic test of predictors of applicant attraction at different stages of the recruiting process. Pers Psychol.

[33] Hausknecht JP, Day DV, Thomas SC (2004). Applicant reactions to selection procedures: an updated model and meta-analysis. Pers Psychol.

[34] Statistics Netherlands: Wat verstaat het CBS onder een allochtoon? https://www.cbs.nl/nl-nl/faq/specifiek/wat-verstaat-het-cbs-onder-een-allochtoon-. Accessed 7 June 2018.

[35] Steiner DD, Gilliland SW (1996). Fairness reactions to personnel selection techniques in France and the United States. J Appl Psychol..

[36] Bauer TN, Truxillo DM, Sanchez RJ, Craig JM, Ferrara P, Campion MA (2001). Applicant reactions to selection: development of the selection procedural justice scale (SPJS). Pers Psychol.

[37] Cohen J (1988). Statistical power analysis for the behavioral sciences.

[38] Nguyen NT, Biderman MD, McDaniel MA (2005). Effects of response instructions on faking a situational judgment test. Int J Sel Assess.

[39] Oostrom JK, Köbis NC, Ronay R, Cremers M (2017). False consensus in situational judgment tests: what would others do?. J Res Pers.

[40] Smither JW, Reilly RR, Millsap RE, Pearlman K, Stoffey RW (1993). Applicant reactions to selection procedures. Pers Psychol.

[41] Ryan AM, Huth M (2008). Not much more than platitudes? A critical look at the utility of applicant reactions research. Hum Resour Manag Rev.

[42] Ryan AM, Greguras GJ, Hakel MD (1998). Life is not multiple choice: reactions to the alternatives. Beyond multiple choice: evaluating alternatives to traditional testing for selection.

[43] Arthur W, Glaze RM, Jarrett SM, White CD, Schurig I, Taylor JE (2014). Comparative evaluation of three situational judgment test response formats in terms of construct-related validity, subgroup differences, and susceptibility to response distortion. J Appl Psychol.

[44] Stumpf H, Stanley JC (1996). Gender-related differences on the college Board's advanced placement and achievement tests, 1982–1992. J Educ Psychol.

[45] Hofstede G (2001). Culture's consequences: comparing values, behaviors, institutions and organizations across nations.

[46] Jetten J, Postmes T, McAuliffe BJ (2002). ‘We're all individuals’: group norms of individualism and collectivism, levels of identification and identity threat. Eur J Soc Psychol.

[47] Ababneh KI, Hackett RD, Schat ACH (2014). The role of attributions and fairness in understanding job applicant reactions to selection procedures and decisions. J Bus Psychol.

[48] College Bescherming Persoonsgegevens (CPB). Gedragscode voor onderzoek & statistiek. 2010. https://www.moaweb.nl/codes-standards/professie/gedragscodes.html. Accessed 7 June 2018.

