# Supplementary material for: Influence of response instructions and response format on applicant perceptions of a situational judgement test for medical school selection
Source: BMC Med Educ. 2018 Nov 26;18:282. doi: 10.1186/s12909-018-1390-0 (PMC6258459; doi:10.1186/s12909-018-1390-0)
Supplement: Supplementary file 1 — SJT applicant perceptions: Microsoft Word Document (.docx): Applicant perception items: The seven items on applicants perceptions that were administered for the four versions of the SJT. (DOCX 14 kb) [file 12909_2018_1390_MOESM1_ESM.docx]

**Additional file 1**

*Applicant perception items*

| Label | Item |
| --- | --- |
| Perceived predictive validity | How would you rate the effectiveness of a Situational Judgement Test for identifying qualified people for medical school? (1: *very ineffective* – 7: *very effective*) |
| Perceived fairness | If you would not be admitted based on a Situational Judgement Test, what would you think of the fairness of this procedure? (1: *very unfair* – 7: *very fair*) |
| Face validity | A Situational Judgement Test is a logical test for identifying qualified applicants for medical school. (1: *strongly disagree* – 7: *strongly agree*) |
| Applicant differentiation | A Situational Judgement Test measures an individual's important qualities, differentiating them from others. (1: *strongly disagree* – 7: *strongly agree*) |
| Study relatedness | A person who scores well on a Situational Judgement Test will be a good medical student. (1: *strongly disagree* – 7: *strongly agree*) |
| Chance to perform | I could really show my skills and abilities through a Situational Judgement Test. (1: *strongly disagree* – 7: *strongly agree*) |
| Ease of cheating | It is easy to cheat or fake on a Situational Judgement Test. (1: strongly disagree – 7: *strongly agree*) |
