# Supplementary material for: Influence of response instructions and response format on applicant perceptions of a situational judgement test for medical school selection
Source: BMC Med Educ. 2018 Nov 26;18:282. doi: 10.1186/s12909-018-1390-0 (PMC6258459; doi:10.1186/s12909-018-1390-0)
Supplement: Supplementary file 2 — SJT applicant perceptions: Microsoft Word Document (.docx): Mean and standard deviations for the four SJT versions: Means (and standard deviations) for process favourability and the other applicant perception items for the four SJT versions and for each subgroup. (DOCX 21 kb) [file 12909_2018_1390_MOESM2_ESM.docx]

**Additional file 2**

*Means (and standard deviations) for process favourability and the other applicant perception items for the four SJT versions.*

|  | Should do rating | Should do pick-one | Would do rating | Would do pick-one |
| --- | --- | --- | --- | --- |
| Process favourability | 4.45 (1.20) | 4.35 (1.31) | 4.47 (1.17) | 4.28 (1.43) |
| Face validity | 4.39 (1.14) | 4.38 (1.41) | 4.33 (1.33) | 4.22 (1.62) |
| Applicant differentiation | 4.45 (1.59) | 3.86 (1.49) | 4.15 (1.45) | 4.02 (1.96) |
| Study relatedness | 3.74 (1.39) | 3.45 (1.34) | 3.70 (1.28) | 3.43 (1.48) |
| Chance to perform | 3.84 (1.57) | 3.41 (1.60) | 3.76 (1.49) | 3.43 (1.64) |
| Ease of cheating | 4.70 (1.85) | 5.15 (1.84) | 5.20 (1.80) | 5.45 (1.78) |

*Means (and standard deviations) for process favourability and the other applicant perception items for the four SJT versions for men and women.*

|  | Should do rating | | Should do pick-one | | Would do rating | | Would do pick-one | |
| --- | --- | --- | --- | --- | --- | --- | --- | --- |
|  | Men | Women | Men | Women | Men | Women | Men | Women |
| Process favourability | 4.29 (1.36) | 4.49 (1.16) | 4.16 (1.35) | 4.43 (1.30) | 4.86 (0.93) | 4.32 (1.23) | 4.10 (1.66) | 4.34 (1.37) |
| Face validity | 4.14 (1.71) | 4.46 (1.26) | 4.43 (1.24) | 4.36 (1.47) | 4.69 (1.26) | 4.19 (1.34) | 3.70 (1.84) | 4.36 (1.54) |
| Applicant differentiation | 4.05 (2.04) | 4.56 (1.26) | 3.71 (1.57) | 3.91 (1.46) | 4.73 (1.34) | 3.93 (1.44) | 3.55 (1.88) | 4.15 (1.62) |
| Study relatedness | 3.95 (1.43) | 3.68 (1.83) | 3.21 (1.41) | 3.54 (1.32) | 4.00 (1.41) | 3.58 (1.21) | 2.95 (1.64) | 3.56 (1.41) |
| Chance to perform | 4.10 (1.64) | 3.76 (1.55) | 3.00 (1.56) | 3.55 (1.60) | 3.96 (1.51) | 3.69 (1.48) | 3.50 (1.82) | 3.41 (1.60) |
| Ease of cheating | 4.86 (1.96) | 4.66 (1.84) | 5.32 (1.84) | 5.09 (1.85) | 4.85 (1.97) | 5.34 (1.72) | 5.95 (1.64) | 5.32 (1.80) |

*Means (and standard deviations) for process favourability and the other applicant perception items for the four SJT versions for first-generation university students and non-first-generation university students.*

|  | Should do rating | | Should do pick-one | | Would do rating | | Would do pick-one | |
| --- | --- | --- | --- | --- | --- | --- | --- | --- |
|  | 1^st^ gen. | non-1^st^ gen. | 1^st^ gen. | non-1^st^ gen. | 1^st^ gen. | non-1^st^ gen. | 1^st^ gen. | non-1^st^ gen. |
| Process favourability | 4.79 (0.95) | 4.25 (1.26) | 3.87 (1.48) | 4.58 (1.14) | 4.61 (1.04) | 4.39 (1.25) | 4.39 (1.45) | 4.21 (1.47) |
| Face validity | 4.93 (1.12) | 4.05 (1.42) | 4.05 (1.31) | 4.52 (1.40) | 4.74 (1.06) | 4.12 (1.40) | 4.26 (1.73) | 4.11 (1.60) |
| Applicant differentiation | 4.46 (1.45) | 4.35 (1.70) | 3.11 (1.29) | 4.16 (1.45) | 4.41 (1.55) | 4.03 (1.36) | 4.11 (1.88) | 3.96 (1.68) |
| Study relatedness | 3.96 (1.32) | 3.60 (1.36) | 3.21 (1.13) | 3.60 (1.35) | 3.89 (1.16) | 3.69 (1.31) | 3.53 (1.68) | 3.36 (1.46) |
| Chance to perform | 4.29 (1.41) | 3.58 (1.56) | 3.11 (1.63) | 3.59 (1.57) | 3.89 (1.42) | 3.64 (1.51) | 3.37 (1.92) | 3.39 (1.57) |
| Ease of cheating | 4.43 (1.45) | 4.92 (1.93) | 5.21 (2.10) | 5.19 (1.76) | 5.26 (1.53) | 5.17 (1.90) | 5.53 (1.84) | 5.49 (1.78) |

*Note.* 1st gen. = first-generation university student non-1^st^ gen. = non-first-generation university student

*Means (and standard deviations) for process favourability and the other applicant perception items for the four SJT versions for applicant of a Dutch, non-Western and Western ethnic background.*

|  | Should do rating | | | Should do pick-one | | |
| --- | --- | --- | --- | --- | --- | --- |
|  | D | NW | W | D | NW | W |
| Process favourability | 4.43 (1.17) | 4.81 (0.99) | 3.64 (1.38) | 4.41 (1.21) | 4.29 (1.29) | 5.00 (1.52) |
| Face validity | 4.20 (1.43) | 5.05 (1.07) | 3.64 (1.21) | 4.38 (1.29) | 4.47 (1.68) | 4.67 (1.51) |
| Applicant differentiation | 4.39 (1.65) | 4.81 (1.44) | 3.55 (1.57) | 3.82 (1.43) | 4.11 (1.52) | 4.50 (1.87) |
| Study relatedness | 3.75 (1.27) | 4.05 (1.40) | 2.91 (1.45) | 3.40 (1.19) | 3.79 (1.55) | 3.83 (1.72) |
| Chance to perform | 3.70 (1.60) | 4.48 (1.21) | 3.09 (1.45) | 3.44 (1.55) | 3.63 (1.67) | 3.50 (1.87) |
| Ease of cheating | 4.89 (1.83) | 4.52 (1.75) | 4.55 (1.86) | 5.21 (1.87) | 5.32 (1.70) | 4.83 (2.14) |
|  | Would do rating | | | Should do pick-one | | |
|  | D | NW | W | D | NW | W |
| Process favourability | 4.43 (1.18) | 4.21 (1.21) | 5.19 (1.07) | 4.37 (1.47) | 4.03 (1.46) | 3.75 (1.19) |
| Face validity | 4.37 (1.33) | 4.06 (1.35) | 4.50 (1.41) | 4.15 (1.63) | 4.28 (1.78) | 3.75 (0.96) |
| Applicant differentiation | 4.00 (1.32) | 4.18 (1.85) | 5.25 (0.71) | 3.97 (1.71) | 4.22 (1.90) | 3.25 (1.26) |
| Study relatedness | 3.65 (1.22) | 3.94 (1.44) | 4.13 (1.25) | 3.48 (1.54) | 3.17 (1.47) | 3.00 (1.16) |
| Chance to perform | 3.65 (1.48) | 3.88 (1.50) | 3.88 (1.55) | 3.47 (1.66) | 3.22 (1.73) | 3.00 (1.16) |
| Ease of cheating | 5.10 (1.83) | 5.53 (1.46) | 5.25 (2.19) | 5.61 (1.76) | 5.17 (2.04) | 5.00 (0.82) |

*Note.* D = Dutch ethnic background NW = Non-Western ethnic background W = Western ethnic background (not Dutch)
